# Cognitive and Behavioural Weaknesses in Children with Reading Disorder and AD(H)D

**DOI:** 10.1038/s41598-019-51372-w

**Published:** 2019-10-23

**Authors:** Sabrina Turker, Annemarie Seither-Preisler, Susanne Maria Reiterer, Peter Schneider

**Affiliations:** 1 Centre for Systematic Musicology, University of Graz, Graz, Austria; 2 Department for Neuroradiology, Section for Biomagnetism, University Hospital Heidelberg, Heidelberg, Germany; 3 BioTechMed Graz, Graz, Austria; 4 Department of Linguistics, University of Vienna, Vienna, Austria

**Keywords:** Occupational health, ADHD

## Abstract

Working memory capacity, an essential prerequisite for language learning and the development of arithmetic skills, has been reported as deficient in children with reading disorder (RD) and attention deficit (hyperactivity) disorder (AD(H)D). However, few studies to date have explored potential associations of working memory impairments and foreign language learning, mathematical skills and school achievement in these groups, in particular in children with a comorbidity of both. In the present study, we assessed working memory, language learning, arithmetic fluency and academic achievement in children (*N* = 166; *M*_*age*_* = *14.3, range 8–18 y), including typically-developing children (n = 89), subjects with RD (*n* = 27), AD(H)D (*n* = 43), and a comorbidity (*n* = 15). While children with AD(H)D performed similar to typically developing children on all tasks, RD children performed weakly on various language learning and working memory tasks, with major deficits in non-word span, phonetic memory and vocabulary learning. Combining weaknesses of the two groups, children with a comorbidity further performed significantly worse on arithmetic skills, learning of sound-symbol combinations and simple digit span forward. The findings suggest a reconsideration of working memory and learning impairments in AD(H)D, while highlighting the additional weaknesses of comorbid children and pointing out severe foreign language learning difficulties in RD children.

## Introduction

Developmental learning disorder with impairments in reading and written expression (synonymous to developmental dyslexia; henceforth RD^1^) is one of the most frequently diagnosed learning disabilities affecting 5–10% of school-aged children^2,3^. Children with RD face severe difficulties during literacy acquisition despite educational opportunity, normal cognitive functioning and intelligence^4^. The most widely accepted theory explaining the underlying difficulties of RD argues in favour of a phonological coding deficit^5^, wherein the key problem lies in a deficient access to phonological representations^6^. Another line of research suggests deficits in statistical learning, a domain-general computational process, which extracts regularities from a quasi-regular system to predict future events^7^. Children with AD(H)D [attention deficit (hyperactivity) disorder], on the other hand, struggle most with attentional problems often accompanied by hyperactivity and/or impulsivity, which frequently lead to social problems and poor academic outcome^8^. Similar to the aetiology of RD, the exact nature and cause of AD(H)D is still under debate (for suggestions, see^9,10^). Although the single profiles of the two developmental disorders may suggest little overlap, between 18 to 45% of children with AD(H)D suffer from RD and vice versa^11^. Various competing hypotheses explaining this comorbidity have been brought forward, many arguing in favour of a shared genetic aetiology in which the neuropsychological profile of comorbid children is an additive combination of the two single diagnostic groups only^12^. Another view is that children with a comorbidity have unique underlying factors in addition to their shared disabilities^13^.

Due to its central importance for controlled effortful processing and learning-related functions^14^, working memory (WM) has been at the core of much research^15^. WM has also been referred to as an individual’s a priori learning potential^16^ given its importance for foreign language learning^17,18^ and the acquisition of arithmetic skills^19^. Baddeley and colleagues^17,20^ were the first who suggested a strong link between verbal WM and novel word learning, promoting it as the word learning device per se^21^. Apart from language learning, also the mastery and integration of simple numerical facts and concepts^22^ and mental arithmetic, the ability to quickly solve basic calculations^23^, relies heavily on WM^24^. Likewise, problems with arithmetic skills have been linked to deficits in WM components, e.g., the central executive^25,26^.

It is thus not surprising that WM deficits have also been found in children with RD^27,28^ and AD(H)D^29^. The severity of reading impairment was found to be significantly associated with WM^30^ and various studies with RD children^31,32^ have reported weaker performance for easy and complex verbal and visuospatial WM tasks as compared to control children. Similarly, about 31.9% of children with AD(H)D have been found to suffer from WM impairments^33^, both in phonological short-term memory, visuospatial WM and executive processes^34,35^. It has been suggested by Kofler *et al*., that reading problems in children with AD(H)D are attributable, at least partly, to underdeveloped working memory^36^ and reading performance is linked to inattention^37^. However, it has also been suggested that only children with AD(H)D younger than 8 years suffer from WM impairments^38^.

Considering the large body of research on WM, comparatively few studies have addressed potential deficits in language learning ability and mental arithmetic in children with RD, AD(H)D and a comorbid diagnosis. Language acquisition impairment (‘developmental language disorder’) and RD frequently co-occur^4,39^ and foreign language learning problems seem to be a natural consequence thereof. However, only few studies have explicitly addressed the association between RD and foreign language acquisition^40–42^, with one study finding that poor language aptitude scores are linked to RD. Although language impairment and AD(H)D also sometimes co-occur^43^, studies reporting impaired language learning skills in children with AD(H)D are mostly limited to pragmatic and social-emotional skills^44,45^. Concerning academic outcomes, highly motivated AD(H)D children perform similar to controls^46^ and problems with academic achievement have been suggested to be more the consequence of behavioural than of cognitive problems^47^. Especially, hyperactivity and impulsivity can negatively impact school grades^48,49^. Pointing towards the problem of comorbidity, children who suffer from reading, math, or spelling deficits in combination with attentional deficits could be much more impaired in learning than children with a mere AD(H)D diagnosis^50^.

Few studies have been conducted with the aim of comparing performances across a variety of tasks between children with RD, AD(H)D and/or a comorbidity. Comparing AD(H)D and RD college students in one study, Sparks *et al*. have shown that AD(H)D and learning-disabled children perform in the average to above-average range on all measures of cognitive ability and academic achievement^51^. In another large study by Gooch *et al*., AD(H)D was characterized by deficits in time perception and executive function, while RD was characterized by phonological problems and weaknesses in duration discrimination^52^. Looking into differences of WM between the two disordered populations, Kibby and Cohen, reported deficits in visuo-spatial WM in AD(H)D children, compared to verbal short-term memory deficits in the RD population^53^. Another study by Willcutt *et al*. found major verbal WM and language difficulties in RD children, while AD(H)D children were only impaired in few measures of reading and verbal WM^12^. In this study, the comorbid groups seemed to be characterized by an additive combination of the profiles of the single diagnosis groups^12^. Helland *et al*. found language impairments in the majority of children with AD(H)D and RD, i.e., a comorbid diagnosis, and in more than 40% of children with only one diagnosis^54^.

To our knowledge, no behavioural studies have compared larger groups of children with different developmental disorders, namely AD(H)D, RD, and a comorbid diagnosis, in a single study looking at a variety of abilities related to memory, language learning and arithmetic skills, as well as academic outcome. We therefore aimed to clarify if children with a comorbid diagnosis of RD and AD(H)D display additive behavioural profiles or even specific weaknesses going beyond those encountered by children with one diagnosis only. We tested performance in WM, language learning, arithmetic tasks and academic achievement in four different populations: typically-developing children (TD), children with RD, children with AD(H)D and children with AD(H)D and RD (comorbid group). We hypothesized that deficits in verbal WM, expected to some extent in all three clinical populations, should be associated with significant impairments in foreign language learning abilities as measured by the LLAMA language aptitude battery^55^ and in mathematical abilities assessed by an arithmetic fluency test^56^ in comparison to the control group. Moreover, we hypothesized that performance in arithmetic fluency and language learning should be positively correlated with academic achievement. In addition, we were interested in general interdependencies of the aforementioned skills in the general population (TD children).

## Material and Methods

### Subjects

We tested a sample of 166 monolingually raised German children and teenagers (103 males/63 females; age range: 8–18 y, *M*_*age*_ = 14.3 ± 1.7 y). The children were categorized into four groups: typically-developing children (TD; *n* = 89, 45 males/44 females; *M*_*age*_ = 14.6 ± 1.6 y), children with RD (*n* = 25, 14 males/11 females; *M*_*age*_ = 13.5 ± 1.5 y), children with AD(H)D (*n* = 37, 29 males/8 females; *M*_*age*_ = 14.3 ± 1.9 y) and children with both RD and AD(H)D (comorbid; *n = *15, all male; *M*_*age*_ = 13.7 ± 2.1 y).

All subjects with disorders were officially diagnosed either by a psychiatrist, psychologist, or a paediatrician. In case of attentional problems, written diagnoses were obtained and subjects with the diagnostic classifications F 90.0/F90.1 (attention deficit hyperactivity disorder/ADHD) or F 98.8 (attention deficit disorder/ADD) according to ICD-10^1^ were included in the study. In order to improve diagnostic accuracies for our sample, the original classifications were re-validated and informal interviews were conducted with the responsible experts to clarify if potential comorbidities had arisen after the initial diagnoses. Children with RD were diagnosed according to the Paediatric Neurology Standards of the University Hospital Heidelberg, using ELFE^57^ for reading comprehension, the ‘Hamburger Schreib-Probe 1–10’^58^ for spelling skills, and H-LAD for phoneme discrimination^59^.

Children with an IQ below 80, as measured with the intelligence test CFT20-R^60^, or any known comorbidities apart from the specified one (e.g. dyscalculia, autism, epilepsy) were excluded from the study. All children were attending usual schools in Germany and had started acquiring their second language English at age 9 ± 1 years. All tested groups were matched as closely as possible with regard to potentially relevant background variables, such as socio-economic status.

### Testing procedure

All testing took place at the University Hospital Heidelberg, Germany, during weekends. Participants received monetary reward for their participation in the study. Parents of participants provided written informed consent before the experiment as approved by the ethic commission of the Medical University Heidelberg. All research was performed in accordance with relevant guidelines and regulations.

### Academic skills

Both parents and their children independently reported the latest school grades (1–5) in German (first language), English (second language/L2) and mathematics in a short interview (children) and a questionnaire (parents).

### Working memory

Subjects performed three verbal, phonological WM tests, namely digit span forward, digit span backward and nonword span. Digit stimuli from the KAI (‘Kurztest für allgemeine Basisgrößen der Informationsverarbeitung’^61^) were used for both the forward and backward task. For the nonword task, German nonwords (e.g, ‘knol’, ‘plax’, ‘bamp’) were created from a syllable database at the Institute of Natural Language Processing, University of Stuttgart, according to German phonotactic rules. For all three tasks, participants had two attempts to correctly repeat the digits/nonwords before another element was added. Per correct repetition, one point was given and a total of 14 points could be achieved in each of the tasks.

### Foreign language learning

Children completed the LLAMA language aptitude battery^55,62^ (tests can be downloaded for free online), consisting of four sub-tests measuring foreign language learning potential. In the ‘Vocabulary learning’ sub-test (LLAMA B) children must learn as many words associated with tiny figures as possible (only visual input) in two minutes. This test assesses their ability to quickly learn vocabulary and form links in memory. The ‘Phonetic memory’ sub-test (LLAMA D) tests the recognition of previously heard words in an unknown language. The children are auditorily presented with words of an unknown language one quickly after the other. Thereafter, they are presented with one word at a time and have to decide for each stimulus if it was part of the sequence presented beforehand or not. In the ‘Sound-symbol correspondence’ sub-test (LLAMA E), children must learn the relationship between 27 simple combinations of digits and letters (e.g., 0í) and the consonant-vowel syllables they correspond to (e.g., 0í corresponds to the syllable/pa/) in two minutes. After two minutes, they are presented with auditory combinations of two pairs of digits and letters (e.g., /patu/) and have to find the correct written form of these (e.g., 0í3é). Finally, in the ‘Grammatical inferencing’ sub-test (LLAMA F), children learn the syntax and semantics of an unfamiliar language in five minutes by being provided with pictures and corresponding sentences. One picture always corresponds to one sentence that gives information about the syntax of the language and the meaning of elements in that sentence. In the testing part afterwards, they are presented with the same pictures and completely new pictures and have to choose the grammatically correct sentence to describe them. For the new pictures, they must have understood the semantic and syntactic rules of the language in order to be capable of choosing the correct sentence. Scores of the LLAMA lie between 0 and 100%.

Additionally, parents were asked how gifted they considered their children for learning foreign languages on a scale from 0 to 10 (parent-reported aptitude). Children had to give the same estimate independently from their parents about themselves (self-reported aptitude).

### Arithmetic competence

Arithmetic competence was assessed by a test designed by Vogel and colleagues, which is based on the French Kit test of arithmetic skills^56^. The test assesses how many calculations a child can correctly perform in a given amount of time (25 seconds for divisions, 50 seconds for easy and complex additions, subtractions and multiplications). Simple calculations included numbers ranging from 1 to 9, while the complex calculations included numbers ranging from 1 to 20. Each correct calculation yielded one point and points were added up to calculate a sum score.

### Inattention, hyperactivity and impulsivity

All parents filled out the FBB-HKS (‘Fremdbeurteilungsbogen für Hyperkinetische Störungen’), which is part of the ‘Diagnostic System for Psychiatric Disorders in Children and Adolescents’ (DISYPS^63^). It assesses symptoms of AD(H)D as specified in DSM V^7^ and ICD 11^1^. Initially planned for the AD(H)D group as a measure of severity only, we assessed these critical variables in all populations to compare results between groups. The questionnaire consists of 20 statements formulated in accordance with the symptom criteria of DSM V, wherein the occurrence of each symptom is rated on a 4-point scale: 0 (never or rarely), 1 (sometimes), 2 (often) and 3 (very often). Altogether, the 20 items assess inattention (10 statements), hyperactivity (6 statements) and impulsivity (4 statements). For further statistical analyses, raw scores were used since for none of the applied test instruments age norms are available.

All statistical calculations were performed with the software package SPSS 25.

## Results

### Correlational analyses

In a first step, the general interdependencies of the assessed variables were analysed in TD children, since this group was considered to be most representative for the normal population; in contrast, in the three disorder groups interdependencies might be distorted by deviations of specific scales from the norm. As some of our tested variables were not normally distributed according to the Kolmogorov-Smirnov-Test, a Spearman’s rho correlation matrix was calculated (Table 1). Results were corrected for multiple comparisons using Benjamini-Hochberg correction^64^.

**Table 1:** Results of the correlational analysis (Spearman’s rho). All assessed scales are included. Correlation coefficients and significance are reported for each correlation.

	Inattention	Hyper-activity	Impulsivity	Maths	German	English	Digit span f	Digit span b	Nonword span	LLAMA B	LLAMA D	LLAMA E	LLAMA F	Arithmetic	Aptitude (s)	Aptitude (p)
Inattention	1.000	**r** = 0.338* **p** = **0.002**	**r** = 0.269* **p** = **0.013**	**r** = 0.330* **p** = **0.003**	r = 0.230 p = 0.039	**r** = 0.252* **p** = **0.022**	r = −0.097 p = 0.393	r = −0.173 p = 0.126	r = −0.187 p = 0.096	r = 0.123 p = 0.268	r = −0.155 p = 0.160	r = −0.057 p = 0.610	r = −0.152 p = 0.169	r = −0.143 p = 0.194	r = −0.146 p = 0.186	r = −0.251 p = 0.033
Hyper-activity		1.000	**r** = 0.548* **p** < **0.001**	r = 0.098 p = 0.390	r = 0.202 p = 0.070	**r** = 0.240* **p** = **0.030**	r = −0.181 p = 0.108	**r** = −0.255* **p** = **0.023**	r = −0.197 p = 0.079	r = −0.169 p = 0.128	r = −0.127 p = 0.253	r = −0.224 p = 0.042	r = 0.011 p = 0.922	r = −0.110 p = 0.318	r = −0.106 p = 337	r = −0.099 p = 0.406
Impulsivity			1.000	r = 0.071 p = 0.532	r = 0.198 p = 0.077	r = 0.229 p = 0.038	r = −0.096 p = 0.397	r = −0.144 p = 0.203	r = −0.175 p = 0.120	r = −0.025 p = 0.824	r = −0.074 p = 0.509	r = −0.132 p = 236	r = −0.017 p = 0.879	r = −0.091 p = 0.411	r = − 0.015 p = 0.890	r = − 0.070 p = 0.561
Maths				1.000	**r** = 0.499* **p** < **0.001**	**r** = 0.303* **p** < **0.001**	r = −0.147 p = 0.207	**r** = −0.391* **p** < **0.001**	r = 0.023 p = 0.844	r = −0.169 p = 0.136	r = −0.219 p = 0.052	r = −0.105 p = 0.359	r = −0.063 p = 0.580	**r** = −0.359* **p** = **0.001**	r = −0.088 p = 0.436	r = 0.054 p = 0.662
German					1.000	**r** = 0.608* **p** < **0.001**	**r** = −0.331* **p** = **0.003**	r = −0.228 p = 0.041	r = −0.126 p = 0.261	r = −0.152 p = 0.166	r = −0.228 p = 0.037	r = −0.219 p = 0.045	r = −0.214 p = 0.05	r = −0.127 p = 0.246	**r** = −0.352* **p** = **0.001**	**r** = −0.371* **p** = **0.002**
English						1.000	r = −0.215 p = 0.053	r = −0.235 p = 0.034	r = −0.097 p = 0.388	r = −0.019 p = 0.860	**r** = −0.271* **p** = **0.012**	**r** = −0.320* **p** = **0.003**	r = − 0.226 p = 0.038	r = −0.136 p = 0.211	**r** = −0.431* **p** < **0.001**	**r** = −0.549* **p** < **0.001**
Digit span f							1.000	**r** = 0.504* **p** < **0.001**	**r** = 0.506* **p** < **0.001**	r = 0.244 p = 0.025	**r** = 0.379* **p** < **0.001**	**r** = 0.444* **p** < **0.001**	**r** = 0.330* **p** = **0.002**	**r** = 0.379* **p** < **0.001**	**r** = 0.341* **p** = **0.002**	**r** = 0.419* **p** < **0.001**
Digit span b								1.000	r = 0.165 p = 0.131	**r** = 0.329* **p** = **0.002**	**r** = 0.339* **p** = **0.002**	**r** = 0.458* **p** < **0.001**	r = 0.203 p = 0.064	**r** = 0.428* **p** < **0.001**	r = 0.236 p = 0.030	r = 0.209 p = 0.085
Nonword span									1.000	r = 0.131 p = 0.236	**r** = 0.268* **p** = **0.014**	r = 0.236 p = 0.030	**r** = 0.334* **p** = **0.002**	r = 0.109 p = 0.320	r = 0.127 p = 0.248	r = 0.236 p = 0.051
LLAMA B										1.000	r = 0.248 p = 0.020	**r** = 0.336* **p** < **0.001**	r = 0.077 p = 0.478	**r** = 0.319* **p** = **0.002**	r = 0.162 p = 0.134	r = 0.174 p = 0.143
LLAMA D											1.000	**r** = 0.323* **p** = **0.002**	r = 0.193 p = 0.071	**r** = 0.285* **p** = **0.007**	r = 0.189 p = 0.080	r = 0.262 p = 0.026
LLAMA E												1.000	**r** = 0.349* **p** = **0.001**	**r** = 0.364* **p** < **0.001**	r = 0.129 p = 0.234	r = 0.223 P = 0.060
LLAMA F													1.000	r = 0.216 p = 0.043	**r** = 0.268* **p** = **0.012**	**r** = 0.372* **p** = **0.001**
Arithmetic														1.000	r = 0.145 p = 0.177	r = 0.200 p = 0.092
Aptitude (s)															1.000	**r** = 0.724* **p** < **0.001**
Aptitude (p)																1.000

*(bold) Significant at p < 0.05 after Benjamini-Hochberg correction computed in R; digit span f = digit span forward; digit span b = digit span backward; aptitude (s) = self-reported aptitude; aptitude (p) = parent-reported aptitude.

Measures of AD(H)D symptom strength according to the FBB-HKS of the DISYPS were included in the correlation matrix, since TD children also frequently scored above 0 on these gradual scales. The FBB-HKS scales were significantly related to each other (inattention vs. hyperactivity: *r* = 0.34, *p* = 0.002, inattention vs. impulsivity: *r* = 0.27, *p* = 0.013). Moreover, inattention was related to poor mathematics grades (*r* = 0.33, *p* = 0.003; please note that a high grade in the German school system signifies poor performance). Hyperactivity was moderately associated with poor English grades (*r* = 0.24, *p* = 0.03) and also with poor performance in the backward span task (*r* = −0.26, *p* = 0.023). Confirming the validity of the language learning test, good (low) English grades were associated with high scores on LLAMA D (*r* = −0.27, *p* = 0.012) and E (*r* = −0.32, *p* = −0.003) as well as self- (*r* = −0.43, *p* < 0.001) and parent-reported language aptitude (*r* = −0.55, *p* < 0.001).

Digit span forward showed striking positive correlations with all language-relevant and all WM measures, as well as with arithmetic fluency score and reported language aptitude.

### Group comparisons

All of the used tests (WM-scales, LLAMA, arithmetic fluency test, DISYPS FBB-HKS) represent well-established published instruments, which, however, are based on raw scores without explicit age norms. Since an ANOVA for the independent variable ‘group’ and the dependent variable ‘age’ was significant (F_(3,165)_ = 3.2, *p* = 0.025; Tukey post hoc comparison for TD [age: 14.6 ± 1.6 y] vs. RD children [age: 13.5 ± 1.5 y], *p* = 0.027), potential age effects might contaminate group-statistical comparisons. In order to exclude this possibility, age was considered as a covariate in the subsequent analyses.

For each of the tested scales we performed a two-way ANCOVA for the independent variables ‘group’ (TD, RD, AD(H)D, comorbid) and ‘sex’ (male, female) with ‘age’ as a covariate (see Table 2). There was a significant effect of ‘age’ on the scores obtained on different scales, again highlighting the necessity to control for age variability. These scales comprised the grade in mathematics (F_(1,133)_ = 4.0, *p* = 0.048), inattention (F_(1,146)_ = 4.6, *p* = 0.033), arithmetic fluency (F_(1,157)_ = 17, *p* < 0.001), LLAMA E (F_(1,157)_ = 10.2, *p* = 0.002), LLAMA F (F_(1,157)_ = 6.7, *p* = 0.01), and digit span forward (F_(1,151)_ = 4.7, *p* = 0.032).

**Table 2:** Behavioural characteristics for all assessed scales of all four groups. Mean values and standard deviations (SD), as well as results of the ANCOVA with age as covariate are reported.

	Group										
	TD		RD		AD(H)D		Comorbid		Main effect for group		
	*M*	*SD*	*M*	*SD*	*M*	*SD*	*M*	*SD*	*F*	*p*	*η*_*p*_ *²*
Digit span forward	7.1_d_	2.1	6.0	2.8	6.9_d_	2.0	4.9_a,c_	1.0	3.7	0.012	0.069
Digit span backward	6.6_b,d_	2.1	5.3_a_	2.2	5.8_d_	1.7	4.1_a.c_	1.5	5.7	0.001	0.102
Nonword span	4.7_b,c,d_	1.6	3.5_a_	1.2	4.0_a_	1.6	3.1 _a_	1.1	7.7	<0.001	0.132
LLAMA B	44.6_b,d_	14.9	32.4_a,c_	18.4	45.7_b,d_	16.9	33.7_a,c_	13.7	6.1	0.001	0.104
LLAMA D	33.7_b,d_	16.0	19.4_a_	14.5	25.7	16.5	20.0_a_	13.8	4.8	0.003	0.084
LLAMA E	59.7_d_	32.0	44.4_d_	32.7	56.2_d_	31.7	24.7_a,b,c_	28.8	4.0	0.008	0.073
LLAMA F	39.6_d_	26.1	30.6	25.2	38.1	26.9	18.0_a_	17.0	2.5	0.063	0.045
Arithmetic competence	145.8_d_	51.4	124.6	30.1	140.5_d_	43.0	100.3_a,c_	26.4	4.7	0.003	0.083
Inattention	4.4_c,d_	4.4	6.9_c,d_	3.8	14.1_a,b_	5.7	13.6_a,b_	4.5	24.7	<0.001	0.337
Hyperactivity	1.1_c,d_	1.9	2.6_c,d_	2.7	5.9_a,b_	4.3	5.8_a,b_	4.3	10.9	<0.001	0.183
Impulsivity	1.0_c,d_	1.5	1.8_c,d_	1.6	4.3_a,b_	3.6	4.7_a,b_	3.4	13.8	<0.001	0.221
Self-reported aptitude	6.3_b,d_	1.5	4.8_a,c_	2.0	6.3_b,d_	1.5	4.0_a,c_	1.9	11.6	<0.001	0.184
Parent-reported aptitude	6.4_b,d_	2.0	4.5_a_	2.0	5.8_d_	2.0	3.7_a,c_	1.2	10.1	<0.001	0.186
Grade Maths	2.6	1.1	2.8	0.9	3.0	1.1	2.9	0.7	0.5	0.669	0.012
Grade German	2.5_b,c,d_	0.8	2.9_a_	0.7	3.1_a_	0.9	3.5_a_	0.6	6.1	0.001	0.108
Grade English	2.7_b,d_	1.0	3.3_a_	0.9	2.8_d_	0.9	3.8_a,c_	0.8	6.5	<0.001	0.114

**Note**. Subscript letters indicate that the mean differs reliably (p < 0.05) from the referred-to mean (Bonferroni-corrected post hoc tests): a = TD group, b = RD group, c = AD(H)D group, d = comorbid group.

Only two ANCOVAs revealed a significant main effect of sex. While grades in German were better among females (F_(1,150)_ = 4.2, *p* = 0.041; males: *M* = 3.1, *SD* = 0.1; females: *M* = 2.6, *SD* = 0.1), hyperactivity was higher among males (F_(1,146)_ = 9.3, *p* = 0.003; males: *M* = 4.1, *SD* = 0.3; females: *M* = 1.8, *SD* = 0.5). For hyperactivity, there was also a group vs. sex interaction (F_(2,146)_ = 6.4, *p* = 0.002), showing that males received higher scores on hyperactivity than females particularly in the RD group (male: *M* = 2.9, *SD* = 0.8; female: *M* = 2.0, *SD* = 0.9) and the AD(H)D group (male: *M* = 6.9, *SD* = 0.6; female: *M* = 2.2, *SD* = 1.1).

**Figure 1:**
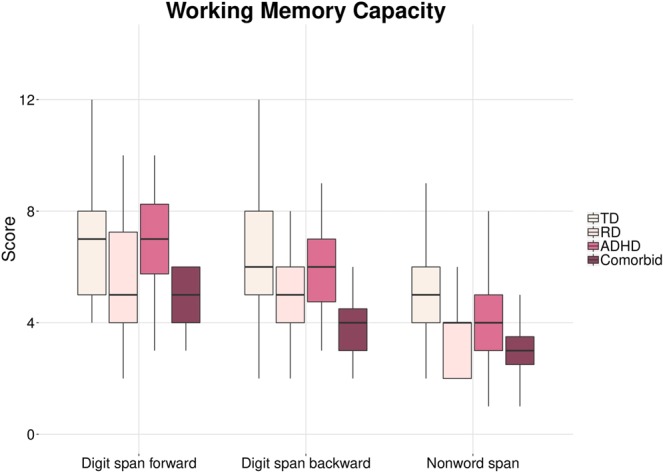
Comparison of working memory measures (digit span forward, digit span backward, nonword span) between the four groups. TD = typically developing children, RD = reading disordered children, AD(H)D = children with attention deficit (hyperactivity) disorder. Score: raw score of respective WM scales.

**Figure 2:**
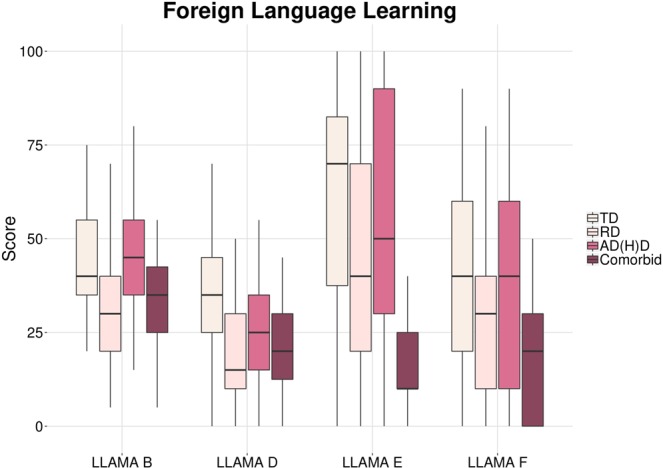
Results of the four tested groups on all four subtests of the LLAMA language aptitude battery. Scores from 0–100 given in %. TD = typically developing children, RD = reading disordered children, AD(H)D = children with attention deficit (hyperactivity) disorder.

Results of the disorder-related group comparisons and post-hoc tests (Tukey HSD) are presented in Figs 1 and 2. Concerning WM, a consistent pattern can be recognized although not all post-hoc comparisons were significant. On all three WM scales, performance was highest in the TD group, slightly lower in the AD(H)D group, again lower in the RD group and lowest in the comorbid group. The following comparisons reached significance: For digit span forward, the comorbid group significantly underperformed both TD (*p* = 0.001) and AD(H)D children (*p* = 0.007). On digit span backward, the comorbid group showed lower performance than both the TD (p = 0.001) and AD(H)D groups (*p* = 0.019) and the RD group performed worse than the TD group (*p* = 0.029). For nonword span, comorbid (*p* < 0.001), RD (*p* = 0.001) and AD(H)D children (*p* = 0.016) all showed impaired performance as compared to TD children (Fig. 1).

Concerning language learning skills as measured by the LLAMA, the same pattern becomes evident, showing a decreasing performance from TD to AD(H)D, RD and comorbid children (Fig. 2). The following post-hoc comparisons reached significance: On the LLAMA B, TD children had significantly higher scores than children with a comorbidity (*p* = 0.027) or RD (*p* = 0.002), but not with AD(H)D. The AD(H)D children significantly outperformed comorbid (*p* = 0.009) and RD children (*p* = 0.001). For LLAMA D, TD children again showed significantly higher performance than comorbid (*p* = 0.007) and RD (*p* = 0.001), but not AD(H)D children. LLAMA E led to poor results only in comorbid children, in contrast to TD (*p* < 0.001), AD(H)D (*p* = 0.004) and RD children (*p* = 0.044). Finally, for LLAMA F, there was a significant difference between TD and comorbid children (*p* = 0.008) and a trend towards the AD(H)D group outperforming the comorbid group (*p* = 0.056). Arithmetic scores differed between TD and comorbid children (*p* = 0.005), as well as AD(H)D and comorbid children (*p* = 0.047).

As expected, for the DISYPS FBB-HKS, AD(H)D and comorbid children (*p* < 0.001), but not RD children, received significantly higher scores than TD controls on impulsivity, hyperactivity and inattention. Moreover, RD children had significantly lower hyperactivity scores compared to AD(H)D (*p* = 0.011) and comorbid children (*p* = 0.001), and also lower scores in inattention and impulsivity (*p* < 0.001). There was a slight trend for RD children to receive higher scores on hyperactivity (*p* = 0.066) and inattention (*p* = 0.079) than TD controls.

Regarding school grades, there were no group differences for maths grades, but TD children had significantly better grades in German than RD children (*p* = 0.025), AD(H)D children (*p* = 0.005) and children with a comorbid diagnosis (*p* < 0.001). For English grades, on the other hand, TD controls had significantly better grades than RD (*p* = 0.004) and comorbid children (*p* = 0.001). Moreover, the difference in English grades between AD(H)D and comorbid children was significant (*p* = 0.003).

### Discriminant analyses

Another major aim of the present study was to investigate which scales were involved in the differentiation between TD children and each of the three diagnostic groups. We therefore performed discriminant analyses, which are shown in Table 3 (combined structural matrices; correlations with the respective discriminant functions below *r* = 0.3 are disregarded). For the differentiation between TD and RD children (*λ* = 0.74, *χ* = 28.8, df = 11, *p* = 0.002; 77.2% of cases correctly classified), the most significant scales for group status distinction were LLAMA D (phonetic memory), LLAMA B (vocabulary learning) and nonword span. The discriminant analysis for TD vs. AD(H)D children (*λ* = 0.470, *χ* = 78.1, df = 11, *p* < 0.001; 85.7% of cases correctly classified) only yielded three significant scale contributions, namely inattention, impulsivity and hyperactivity. Although this is not really surprising, as it confirms that the AD(H)D group did not show any comorbidities and it also shows that there were only minor other problems (e.g. slight WM deficits in one of the three assessed dimensions that only reached significance in the ANCOVA analyses) in this group. The third discriminant analysis differentiated between TD and comorbid children (*λ* = 0.495, *χ* = 60.1, df = 11, *p* < 0.001; 93.3% of cases correctly classified), revealing that all scales except for LLAMA B and D were involved in the differentiation between these groups.

**Table 3:** Results of discriminant analyses for TD children vs. the three disordered populations (from left to right: RD, AD(H)D, comorbid children).

Structural Matrix			
	TD vs. RD	TD vs. AD(H)D	TD vs. comorbid
LLAMA B	0.524		
LLAMA D	0.605		
LLAMA E	0.336		−0.384
LLAMA F			−0.326
Digit span forward	0.363		−0.402
Digit span backward	0.454		−0.456
Nonword span	0.585		−0.388
Inattention	−0.405	0.849	0.723
Hyperactivity	−0.495	0.726	0.704
Impulsivity	−0.408	0.615	0.689
Arithmetic fluency	0.354		−0.357

Correlations with the respective discriminant functions <0.3 are not shown in the table.

**Figure 3:**
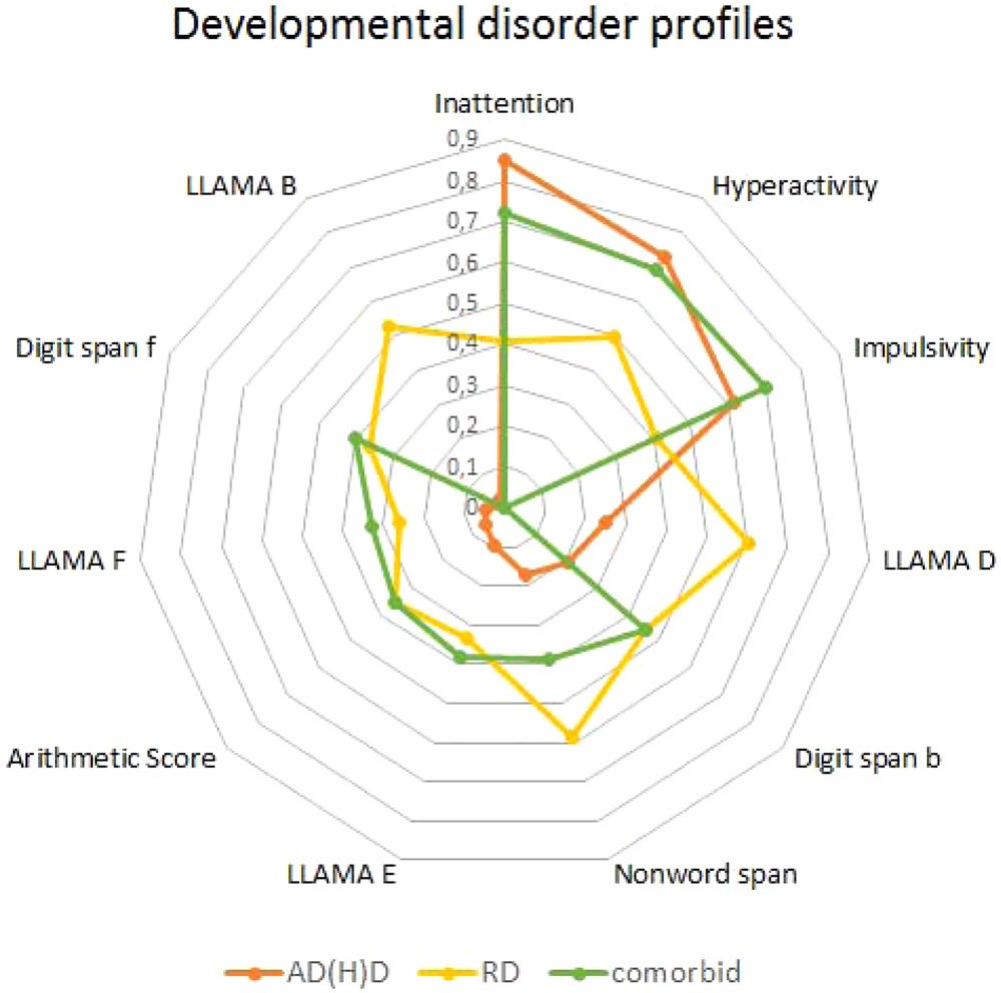
Results of the discriminant analyses for the four tested groups including all tested scales. The graph shows the major contributions of scales differentiating between TD and AD(H)D children (orange), TD and RD children (yellow) and TD and comorbid children (green).

Results show that the three attentional problem scales (inattention, hyperactivity, impulsivity) are most closely associated with AD(H)D status, whereas RD group status is primarily associated with differences in phonological short-term memory, vocabulary learning and phonetic memory. The differentiation between TD and comorbid children shows that the profile of the latter is at least an addition of the profiles of other two diagnostic groups (Fig. 3).

## Discussion

The results of the present study highlight that despite the frequent comorbidity between AD(H)D and RD, the cognitive and behavioural profiles of the two diagnostic groups are quite distinct, if not even dissimilar. While children with AD(H)D showed practically no cognitive impairments, children with RD displayed numerous weaknesses in cognitive performance affecting foreign language learning, WM capacity and academic achievement. Behavioural problems, on the other hand, were strongly associated with AD(H)D status and were also the most significant characteristics of children with a comorbidity of RD and AD(H)D. The results further indicate that comorbid children do not only exhibit the behavioural and cognitive weaknesses present in the single diagnosis groups, but also struggle with additional difficulties, such as the acquisition of arithmetic skills.

### Profile of children with AD(H)D

Numerous studies have proposed and tested WM trainings to improve WM performance and AD(H)D symptoms in affected individuals^65,66^. While we agree that WM might generally be ameliorated through training, it should be pointed out that studies on deficient WM in AD(H)D have yielded quite inconsistent results^67^. It thus remains questionable if improving WM in individuals with AD(H)D does in any way help to improve cognitive functions or behavioural problems. In the present study, we only found minor weaknesses in phonological WM (nonword span).

Furthermore, AD(H)D children performed on average in all other tasks assessing language learning, school achievement (except for English grades) and arithmetic skills. This is not in line with the view that pure AD(H)D (without an additionally diagnosed comorbidity) is typically accompanied by problems in language learning, reading and arithmetic^68^, affecting school achievement^69^. While our finding is not really surprising in view of the a priori group classifications of AD(H)D vs. RD vs. comorbid children, it is not self-evident since subtle impairments in other domains may remain below the threshold of a psychological/clinical diagnoses and hence may be overlooked.

We therefore conclude that children and adolescents with AD(H)D show no severe cognitive impairments and are not necessarily limited in their school achievement, although this is a problem of circularity within the diagnosis of ADHD. A clinical diagnosis according to DSM is based on the presence of functional and behavioural impairments, affecting performance at school and at home. As a result, it would be little surprising to detect poor academic achievement in AD(H)D children as they partly might have received their diagnosis because of these issues. The results of our study hint towards the fact that behavioural and academic problems are primarily due to inattention, hyperactivity and impulsivity, instead of cognitive problems. This is further supported by findings of the discriminant analysis showing that merely scores for inattention, hyperactivity and impulsivity (FBB-HKS) could differentiate children with AD(H)D from TD children in more than 90% of cases.

While cognitive and WM impairments were hardly present, the findings of the behavioural questionnaire suggest that AD(H)D symptoms undergo very little change through adolescence. All AD(H)D children of the present study were between 13–17 years at the time we assessed them for the present study, and they had been diagnosed by a clinician before age 12. Therefore, results in the FBB-HKS show that they were still experiencing problems with inattention, hyperactivity and impulsivity as observed by their parents, placing them again in the AD(H)D diagnostic group many years after their initial diagnosis. This favours the assumption that AD(H)D is a life-long condition that undergoes little change during puberty. Another conclusion from these results is that parent-rating scales support clinical diagnoses and are trustworthy additional tools for assessment.

It is particularly interesting that inattention was highly associated with poor mathematics grades in TD children, although there were no significant differences in mathematics performance at school between all three disordered populations and TD children. If scores in inattention are particularly linked to school grades in mathematics, this should also be reflected in in children with AD(H)D and those with a comorbid diagnosis. However, this was not the case. Interestingly, inattention was also not linked to German or English grades in the correlational analyses, further questioning if pure behavioural problems with inattention are really severely impacting academic performance.

The lack of substantial cognitive impairments in AD(H)D children suggests that we need to be very cautious of treating individuals with AD(H)D as a truly ‘disordered’ population and proposing different treatments (e.g., WM training) to ameliorate cognitive functions. We agree that AD(H)D should rather be seen as “a constellation of personality traits and cognitive styles that cluster, in pure form, in a relatively small number of people and in various combinations and permutations in large numbers of people”^70^. This could certainly help loosen the stigma surrounding this developmental ‘disorder’. Moreover, the major focus in schools and educational settings should be put on helping children with AD(H)D to fully develop their potentials, of which there are many, despite their problems with inattention, hyperactivity and impulsivity.

### Profile of children with RD

It has been suggested that “[in]attention may precede reading difficulty and adversely affect the child’s capacity to profit from academic instruction”(^71^, p.29), resulting in poor academic achievement in RD. In the present study, the RD group received very poor scores on a variety of scales, in particular WM tasks (nonword span, digit span backward) and language learning tasks. When looking at the mean scores archived in the tasks (Table 1) and the visualizations provided (Figs 1–3), it is quite clear that even if some comparisons did not reach statistical significance, the RD group underperformed not only TD children in a large variety of tasks, but often also AD(H)D children. However, they did not exhibit major problems with inattention, hyperactivity or impulsivity. As a result, we cannot support the claim that their academic problems are mainly due to short attention span or concentration problems. Rather, we argue that their linguistic problems, as found in the variety of language tasks administered, are associated with poor performance in subjects at school where reading, writing and language learning are demanded.

According to the results of the discriminant analysis, nonword span, phonetic memory and vocabulary learning were most significantly associated with RD group status, closely followed by other WM tests and the arithmetic test. It is not a surprise that nonword span is linked to RD since language impairments and RD have both been associated with poor phonological short-term memory^27,28^. However, the phonetic memory test does in no form include reading or writing but is based on auditory input only. The major task in this test is to listen to speech input without explicitly memorizing it. This finding therefore clearly supports a WM-related auditory^10^ and/or phonological deficit of individuals with RD. Compared to nonword span, the phonetic memory task is very similar, but no repetition is involved, so no speech motor action is required.

It was further surprising that RD children had deficits in vocabulary learning, but not in analytic language learning tasks. It seems that those tasks involving WM (LLAMA B and D) were more severely affected than those requiring analytical thinking (LLAMA E and F), although mean values were still much lower than in TD children. This hints towards the possibility that children with RD do not suffer from weaknesses in analytical thinking or problem-solving but rather display weaknesses in phonetic or phonological tasks in combination with WM.

### Profiles of children with AD(H)D and RD

High rates of comorbidity have been found not only in our current study, where about a fifth of all AD(H)D children suffered from a comorbidity, mostly RD (N = 15), dyscalculia or autism (leading to exclusion), but also in other studies^11^. Given the lack of cognitive impairments we found in AD(H)D children compared to RD children, we propose a clear behavioural distinction between these two diagnostic groups. The results of the discriminant analyses support the claim that there is very little overlap between the two, while the profile for children with a comorbidity displays a combination or even intensification of the single diagnostic profiles.

Statistical analyses revealed that comorbid children were significantly impaired on all measures (all WM scales, school grades, arithmetic fluency, all language learning tasks), and even in tasks where both the RD and AD(H)D groups performed similar to the TD group (e.g., arithmetic fluency, sound-symbol association, digit span forward). According to the results of the discriminant analysis, a differentiation between typical and comorbid group status is closely associated with the presence of inattention, hyperactivity and impulsivity, accompanied by linguistic and mathematical deficits. Although our findings are partly in accordance with the shared genetic aetiology theory^9,12^, they suggest that AD(H)D symptoms are more indicative of comorbid group status than RD symptoms in contrast to results of other studies^72^. So it seems that limitations in attention span together with concentration problems are severely affecting children with a comorbid diagnosis. Moreover, our findings provide evidence that additional weaknesses, e.g. in the arithmetic domain, may occur. Such children may suffer from further-reaching deficits, e.g., in overall brain development, which could explain their weaker performance in different cognitive domains. Therefore, the development of comorbid children should be individually monitored with close attention.

### The links between working memory, arithmetic skills and language learning

Correlational analyses of TD children revealed strong links between WM scales, all measures of foreign language learning ability and arithmetic skills. These findings support the claim that WM is an essential shared foundation of these two abilities. This is in accordance with intelligence research, showing that a high WM capacity is an essential part of domain-unspecific general intelligence^73^. It is thus not really surprising that our findings, along with others^20,23^, show that WM is a fundamental factor associated with both language learning ability and arithmetic fluency. This finding, in turn, suggests that WM deficits have an impact on the development of learning disabilities. The strong correlation between arithmetic fluency and language learning stands in contrast to the common belief that language and mathematical skills are rarely associated. Rather, the findings of the present study suggest that high language learning ability goes hand in hand with high arithmetic skill, possibly even beyond the mere contribution of high WM capacity to both.

Interestingly, the grades for native and foreign language correlated with almost all WM scores, but not with vocabulary learning ability. This is particularly surprising since foreign language classes focus on and foster vocabulary learning, and it would thus be logical to assume that children who learn vocabulary easily have better grades.

One of the most striking correlations in TD children was that of self- and parent-reported foreign language learning aptitude and their relation to school grades. The high correlation between self- and parent-reported language aptitude signifies a high validity of these measures. With regard to school grades, the question of causality is of particular interest: Do children think of themselves as less gifted because of their negative school experience in learning languages or do they have bad grades because of their underlying lack of potential for learning foreign languages? The same question applies to the parent-reported aptitude. From the present study, we cannot answer this question but both options as well as a combination or interaction of both are possible.

## Conclusion

Overall, children with RD or a comorbidity of RD and AD(H)D considered themselves less gifted for learning foreign languages (in accordance with their parents’ view), struggled with various aspects of language learning and underperformed TD and AD(H)D children in almost all tasks related to WM and language learning. RD was closely associated with poor performance in nonword span, digit span backward, phonetic memory and vocabulary learning, but not with behavioural problems. In contrast, children with AD(H)D were basically characterised by problems with inattention, hyperactivity, and impulsivity that were associated with only marginal impairments in language-related WM (non-word span). Comorbid children suffered from all indicators of RD and AD(H)D and additionally had difficulties with arithmetic fluency. Concerning academic achievement, RD and comorbid children received poor grades both in German and English, while AD(H)D children only exhibited a weak performance in English. Overall, we suggest that RD is associated with cognitive but not behavioural difficulties, while AD(H)D is strongly linked to behavioural, but not to cognitive weaknesses. This suggests that it is of vital importance to further investigate the competence profile of comorbid children, as other skills not considered among the classical weaknesses of AD(H)D and RD might be impaired. That way, therapies and learning strategies may be individually adjusted to the needs of each child.

## Data Availability

The datasets generated during and/or analysed during the current study are available from the corresponding author on reasonable request.

## References

[1] World Health Organization. International statistical classification of diseases and related health problems (11th Revision), https://icd.who.int/browse11/l-m/en (2018).

[2] Siegel LS (2006). Perspectives on dyslexia. Paediatrics & child health.

[3] Turker, S. Exploring the neurofunctional underpinning of developmental dyslexia: a review focusing on dyslexic children in *The* Talking *Species* (ed. Marin, M. & Luef, E.) 337–377 (Uni Graz Press, 2018).

[4] Bishop DVM, Snowling MJ (2004). Developmental Dyslexia and Specific Language Impairment: Same or Different?. Psychological Bulletin.

[5] Ramus F, White S, Frith U (2006). Weighing the evidence between competing theories of dyslexia. Developmental Science.

[6] Ramus F, Szenkovits G (2008). What Phonological Deficit?. Quarterly Journal of Experimental Psychology.

[7] Schmalz X, Altoè G, Mulatti C (2017). Statistical learning and dyslexia: A systematic review. Annals of Dyslexia.

[8] Diagnostic and statistical manual of mental disorders. (American Psychiatric Association, 2017).

[9] Willcutt E, Doyle A, Nigg J, Faraone S, Pennington B (2005). Validity of the Executive Function Theory of Attention-Deficit/Hyperactivity Disorder: A Meta-Analytic Review. Biological Psychiatry.

[10] Serrallach, B. *et al*. Neural Biomarkers for Dyslexia, ADHD, and ADD in the Auditory Cortex of Children. *Frontiers in Neuroscience* **10**, 10.3389/fnins.2016.00324 (2016). 10.3389/fnins.2016.00324 PMC4945653 27471442

[11] Germanò E, Gagliano A, Curatolo P (2010). Comorbidity of ADHD and Dyslexia. Developmental Neuropsychology.

[12] Willcutt E, Pennington B, Olson R, Chhabildas N, Hulslander J (2005). Neuropsychological Analyses of Comorbidity Between Reading Disability and Attention Deficit Hyperactivity Disorder: In Search of the Common Deficit. Developmental Neuropsychology.

[13] Moura O (2017). Neurocognitive functioning in children with developmental dyslexia and attention-deficit/hyperactivity disorder: Multiple deficits and diagnostic accuracy. Journal of Clinical and Experimental Neuropsychology.

[14] Conway A (1996). Individual Differences in Working Memory Capacity: More Evidence for a General Capacity Theory. Memory.

[15] Baddeley, A. The concept of working memory: A view of its current state and probable future development in *Exploring Working Memory: Selected Works of Alan Baddeley* (ed. Baddeley, A.) 99–106 (Routledge/Taylor & Francis, 2017).

[16] Alloway T, Alloway R (2010). Investigating the predictive roles of working memory and IQ in academic attainment. Journal of Experimental Child Psychology.

[17] Baddeley A, Gathercole S, Papagno C (1998). The phonological loop as a language learning device. Psychological Review.

[18] Wen Z, Biedroń A, Skehan P (2016). Foreign language aptitude theory: Yesterday, today and tomorrow. Language Teaching.

[19] Menon V (2016). Working memory in children’s math learning and its disruption in dyscalculia. Current Opinion in Behavioral Sciences.

[20] Baddeley A (2003). Working memory: looking back and looking forward. Nature Reviews Neuroscience.

[21] Wen, Z. Working Memory and Second Language Learning: Towards an Integrated Approach. (Multilingual Matters, 2016).

[22] Dehaene S (2001). Precis of The Number Sense. Mind and Language.

[23] Lyons I, Price G, Vaessen A, Blomert L, Ansari D (2014). Numerical predictors of arithmetic success in grades 1-6. Developmental Science.

[24] Adams, J. & Hitch, G. Children’s mental arithmetic and working memory in *The Development of Mathematical* Skills (ed. Donlan, C.) 153–173 (Psychology Press, 1998).

[25] Andersson U, Lyxell B (2007). Working memory deficit in children with mathematical difficulties: A general or specific deficit?. Journal of Experimental Child Psychology.

[26] Passolunghi M, Siegel L (2004). Working memory and access to numerical information in children with disability in mathematics. Journal of Experimental Child Psychology.

[27] Moll K, Göbel S, Gooch D, Landerl K, Snowling M (2014). Cognitive Risk Factors for Specific Learning Disorder. Journal of Learning Disabilities.

[28] Schuchardt K, Maehler C, Hasselhorn M (2008). Working Memory Deficits in Children with Specific Learning Disorders. Journal of Learning Disabilities.

[29] Martinussen R, Tannock R (2006). Working Memory Impairments in Children with Attention-Deficit Hyperactivity Disorder with and without Comorbid Language Learning Disorders. Journal of Clinical and Experimental Neuropsychology.

[30] Gathercole S, Alloway T, Willis C, Adams A (2006). Working memory in children with reading disabilities. Journal of Experimental Child Psychology.

[31] Swanson H (1999). Reading Comprehension and Working Memory in Learning-Disabled Readers: Is the Phonological Loop More Important Than the Executive System?. Journal of Experimental Child Psychology.

[32] Swanson H, Zheng X, Jerman O (2009). Working Memory, Short-Term Memory, and Reading Disabilities. Journal of Learning Disabilities.

[33] Fried R (2016). Clinical correlates of working memory deficits in youth with and without ADHD: A controlled study. Journal of Clinical and Experimental Neuropsychology.

[34] Kasper L, Alderson R, Hudec K (2012). Moderators of working memory deficits in children with attention-deficit/hyperactivity disorder (ADHD): A meta-analytic review. Clinical Psychology Review.

[35] Martinussen R, Hayden J, Hogg-Johnson S, Tannock R (2005). A meta-analysis of working memory impairments in children with attention-deficit/hyperactivity disorder. Journal of the American academy of child & adolescent psychiatry.

[36] Kofler M (2019). Do working memory deficits underlie reading problems in attention-deficit/hyperactivity disorder (ADHD)?. Journal of Abnormal Child Psychology.

[37] Plourde V (2018). Cognitive mechanisms underlying the associations between inattention and reading abilities. Developmental neuropsychology.

[38] Sowerby P, Seal S, Tripp G (2010). Working Memory Deficits in ADHD. Journal of Attention Disorders.

[39] Catts H, Adlof S, Hogan T, Weismer S (2005). Are Specific Language Impairment and Dyslexia Distinct Disorders?. Journal of Speech, Language, and Hearing Research.

[40] Chung K, Ho C (2009). Second Language Learning Difficulties in Chinese Children with Dyslexia: What are the Reading-Related Cognitive Skills that Contribute to English and Chinese Word Reading?. Journal of Learning Disabilities.

[41] Downey D, Snyder L, Hill B (2000). College students with dyslexia: persistent linguistic deficits and foreign language learning. Dyslexia.

[42] Ho C, Fong K (2005). Do Chinese Dyslexic Children Have Difficulties Learning English as a Second Language?. Journal of Psycholinguistic Research.

[43] Cohen N (2000). The Interface between ADHD and Language Impairment: An Examination of Language, Achievement, and Cognitive Processing. Journal of Child Psychology and Psychiatry.

[44] Geurts H, Embrechts M (2008). Language Profiles in ASD, SLI, and ADHD. Journal of Autism and Developmental Disorders.

[45] Staikova E, Gomes H, Tartter V, McCabe A, Halperin J (2013). Pragmatic deficits and social impairment in children with ADHD. Journal of Child Psychology and Psychiatry.

[46] Gut J, Heckmann C, Meyer C, Schmid M, Grob A (2012). Language skills, mathematical thinking, and achievement motivation in children with ADHD, disruptive behavior disorders, and normal controls. Learning and Individual Differences.

[47] Lucangeli D, Cabrele S (2006). Mathematical Difficulties and ADHD. Exceptionality.

[48] Barry T, Lyman R, Klinger L (2002). Academic Underachievement and Attention-Deficit/Hyperactivity Disorder. Journal of School Psychology.

[49] Merrell C, Tymms P (2001). Inattention, hyperactivity and impulsiveness: Their impact on academic achievement and progress. British Journal of Educational Psychology.

[50] Mayes S, Calhoun S (2007). Learning, Attention, Writing, and Processing Speed in Typical Children and Children with ADHD, Autism, Anxiety, Depression, and Oppositional-Defiant Disorder. Child Neuropsychology.

[51] Sparks R, Javorsky J, Philips L (2005). Comparison of the Performance of College Students Classified as ADHD, LD, and LD/ADHD in Foreign Language Courses. Language Learning.

[52] Gooch D, Snowling M, Hulme C (2010). Time perception, phonological skills and executive function in children with dyslexia and/or ADHD symptoms. Journal of Child Psychology and Psychiatry.

[53] Kibby MY, Cohen MJ (2008). Memory functioning in children with reading disabilities and/or attention deficit/hyperactivity disorder: a clinical investigation of their working memory and long-term memory functioning. Child Neuropsychology.

[54] Helland W, Posserud M, Helland T, Heimann M, Lundervold A (2016). Lanugage Impairments in Children with AD(H)D and in Children with Reading Disorder. Journal of Attention Disorders.

[55] Meara, P. LLAMA Language Aptitude Tests: The Manual. (Lognostics, 2005).

[56] Vogel S (2017). Processing the order of symbolic numbers: A reliable and unique predictor of arithmetic fluency. Journal of Numerical Cognition.

[57] Lenhard, W. & Schneider, W. ELFE 1–6: ein Leseverständnistest für Erst- bis Sechstklässler. (Hogrefe, 2006).

[58] May, P. *Hamburger Schreib-Probe 1–10*. (Verlag für Pädagogische Medien, 2012).

[59] Brunner M (2008). Heidelberg Phoneme Discrimination Test (HLAD): Normative Data for Children of the Third Grade and Correlation with Spelling Ability. Folia Phoniatrica et Logopaedica.

[60] Weiß, R. *Grundintelligenztest Skala 2 - Revision*. (Hogrefe, Verl. für Psychologie, 2006).

[61] Lehrl, S., Gallwitz, A., Blaha, L. & Fischer, B. Kurztest für Allgemeine Intelligenz. (Vless, 1992).

[62] Rogers V, Meara P, Barnett-Legh T, Curry C, Davie E (2017). Examining the LLAMA aptitude tests. Journal of the European Second Language Association.

[63] Brühl B, Döpfner M, Lehmkuhl G (2000). Der Fremdbeurteilungsbogen für hyperkinetische Störungen (FBB-HKS) - Prävalenz hyperkinetischer Störungen im Elternurteil und psychometrische Kriterien. Kindheit und Entwicklung.

[64] Benjamini Y, Hochberg Y (1995). Controlling the False Discovery Rate: A Practical and Powerful Approach to Multiple Testing. Journal of the Royal Statistical Society: Series B (Methodological).

[65] Klingberg T (2005). Computerized Training of Working Memory in Children With ADHD-A Randomized, Controlled Trial. Journal of the American Academy of Child & Adolescent Psychiatry.

[66] Beck S, Hanson C, Puffenberger S, Benninger K, Benninger W (2010). A Controlled Trial of Working Memory Training for Children and Adolescents with ADHD. Journal of Clinical Child & Adolescent Psychology.

[67] Melby-Lervåg M, Hulme C (2013). Is working memory training effective? A meta-analytic review. Developmental Psychology.

[68] Andreou G, Agapitou P, Karapetsas A (2005). Verbal skills in children with ADHD. European Journal of Special Needs Education.

[69] Frazier T, Youngstrom E, Glutting J, Watkins M (2007). ADHD and Achievement. Journal of Learning Disabilities.

[70] Gualtieri C, Johnson L (2005). ADHD: Is objective diagnosis possible?. Psychiatry (Edgmont).

[71] August G, Garfinkel B (1990). Comorbidity of ADHD and reading disability among clinic-referred children. Journal of Abnormal Child Psychology.

[72] Pennington B, Groisser D, Welsh M (1993). Contrasting cognitive deficits in attention deficit hyperactivity disorder versus reading disability. Developmental Psychology.

[73] Stern, E. & Neubauer, A. Intelligenz - Große Unterschiede und Ihre Folgen. (DVA, 2013).

